# Photothermal excitation efficiency enhancement of cantilevers by electron beam deposition of amorphous carbon thin films

**DOI:** 10.1038/s41598-020-74433-x

**Published:** 2020-10-15

**Authors:** Marcos Penedo, Ayhan Yurtsever, Keisuke Miyazawa, Hirotoshi Furusho, Kiyo-Aki Ishii, Takeshi Fukuma

**Affiliations:** 1 Nano Life Science Institute (WPI-NanoLSI), Kanazawa University, Kanazawa, 920-1192 Japan; 2 Division of Electric Engineering and Computer Science, Kanazawa University, Kakuma-machi, Kanazawa, 920-1192 Japan; 3 Faculty of Frontier Engineering, Kanazawa University, Kakuma-machi, Kanazawa, 920-1192 Japan; 4 Department of Integrative Medicine for Longevity, Graduate School of Medical Sciences, Kanazawa University, Kanazawa, 920-8640 Japan

**Keywords:** Imaging techniques, Techniques and instrumentation, Atomic force microscopy, Scanning probe microscopy, Atomic force microscopy, Scanning probe microscopy, Imaging techniques, Atomic force microscopy, Molecular imaging, Atomic force microscopy, Scanning probe microscopy

## Abstract

In recent years, the atomic force microscope has proven to be a powerful tool for studying biological systems, mainly for its capability to measure in liquids with nanoscale resolution. Measuring tissues, cells or proteins in their physiological conditions gives us access to valuable information about their real ‘in vivo’ structure, dynamics and functionality which could then fuel disruptive medical and biological applications. The main problem faced by the atomic force microscope when working in liquid environments is the difficulty to generate clear cantilever resonance spectra, essential for stable operation and for high resolution imaging. Photothermal actuation overcomes this problem, as it generates clear resonance spectra free from spurious peaks. However, relatively high laser powers are required to achieve the desired cantilever oscillation amplitude, which could potentially damage biological samples. In this study, we demonstrate that the photothermal excitation efficiency can be enhanced by coating the cantilever with a thin amorphous carbon layer to increase the heat absorption from the laser, reducing the required excitation laser power and minimizing the damage to biological samples.

## Introduction

Microcantilever based sensors have demonstrated to be useful tools for the detection of tiny forces or masses^1,2^. In these systems, an efficient and clean excitation of the microcantilevers' resonances is essential for achieving high sensitivity and reproducibility. An example of a cantilever-based device is the atomic force microscope (AFM), which has become a successful tool in biological and medical studies for its capability to obtain nanoscale resolution when working in liquid physiological conditions.

The most common AFM techniques to measure biomaterials are grouped in what is known as dynamic mode AFM^3,4^, where the cantilever is mechanically oscillated at or near its resonance frequency. In order to induce mechanical vibration, the acoustic excitation^5^ is the typical method applied, but when working in liquids, this actuation technique introduces spurious peaks in the resonance spectra^6^. Some magnetic based excitation methods were developed to overcome this problem, such as magnetic torque^7^, magnetic gradient^8–10^ or magnetostriction^11^; where an alternating magnetic field induces the mechanical oscillation of the cantilever, producing clean resonance spectra. As magnetic field generators are based in coils, the higher the frequency, the higher the impedance, increasing therefore the amount of heat generated by the current driven through the coils. As a result, this effect could damage biological samples, for example, when exciting high resonance modes in multifrequency AFM^12^ or when using the high frequency cantilevers required for high speed AFM^13,14^. In addition, the implementation of the required coil in the AFM head complicates the installation of other components, such as optical microscopes for fluorescence or confocal microscopy; and some magnetic coatings may be toxic for biological samples when corroding in liquid media.

Other techniques based on electric excitation such as piezoelectric^15^, electrothermal^16,17^, and Lorentz force^18^ require complexly designed microfabricated cantilevers, compared to electrostrictive^19^ and electrostatic^20^ excitation methods, which only require metal coatings on a cantilever. However, the application of a bias voltage in liquid can generate surface charges diffusion or uncontrolled electrochemical reactions, hampering its use in liquids.

Over the past years, a new excitation technique, known as photothermal actuation^21^, is gaining importance since it provides a wide frequency bandwidth excitation and clean cantilever resonance spectra. In the photothermal actuation, a metal coated cantilever is irradiated with a power-modulated excitation laser focused close to the cantilever clamping area. The thermal expansion originated by the excitation laser on the metal-coating induces mechanical stress in the cantilever, provoking the cantilever oscillation. Nevertheless, due to its intrinsic low excitation efficiency especially for high resonance modes^22^, relatively high laser power (in the order of tens of milliwatts) is required to achieve the desired cantilever oscillation amplitude, which can potentially result in a rise in liquid and/or sample temperature, becoming a potential problem for certain biological samples. This effect becomes more notorious when high spring constant cantilevers are used. In order to increase the photothermal excitation efficiency, it is crucial to maximize the heat absorption from the laser on the cantilever, minimizing the laser reflection on the cantilever surface. Fresnel equation is used to calculate the reflectivity *R* in an interface between two different mediums considering normal incidence:

$$ R = \left[ {\frac{{n_{2} - n_{1} }}{{n_{2} + n_{1} }}} \right]^{2} $$

where $n_{1}$ and $n_{2}$ are the refraction indexes of medium 1 and 2, respectively. Typical cantilevers used in AFM for photothermal excitation are gold coated. Considering our custom-built system whose excitation laser has a wavelength $\lambda$ = 785 nm, the refraction indexes for water and gold are $n_{1}$ = 1.32^23^ and $n_{2}$ = 0.12^24^. Replacing these values in the Fresnel equation leads to a R = 0.69, which means that more than two-thirds of the radiation is reflected back to the water. Less than one-third of the laser power is transmitted and absorbed by the gold film, transformed into heat, harnessing a low amount of the laser power into cantilever oscillation. Different approaches can be found to overcome this problem. For example, it is possible to further improve the photothermal efficiency by using trapezoidal-shaped cross section cantilevers, which display a higher photothermal efficiency compared to rectangular-shaped cross section due to difference in thermal distribution in the cantilever^25^, but the efficiency still remains low. Also, a short wavelength laser beam may improve the excitation efficiency^26^, but a short wavelength light may damage biological samples or organic molecules. Other approaches take advantage of other physical phenomena to increase the laser absorption, such as placing gold nanoparticles (Au NPs) on cantilevers to excite the plasmon resonance on the Au NPs, which increases the laser absorption and hence the temperature of the cantilever, improving the photothermal excitation efficiency^27^. However, this last method requires tedious tuning of the Au NPs size and shape, and NPs deposition on the cantilever, which is different for each cantilever type, resulting in a complicated cantilever preparation.

Although the previous approaches slightly improve the photothermal efficiency, the most promising path to increase it is one consequence of the Fresnel equation: coating the cantilever with a high refraction index material $n_{2}$ increases the denominator $n_{2} + n_{1}$ in the Fresnel equation and consequently minimizes the reflectivity *R*, rising the amount of laser power absorbed by the cantilever, and increasing the cantilever excitation efficiency. Au/Pt films have been already evaluated as cantilever coating^28^, but this material still keeps a high reflectivity for infrared wavelengths^29^, not being suitable for delicate bio samples. Colloidal graphite was also tested as a photothermal absorption layer^30^; however, the colloidal graphite coating is deposited manually with a micromanipulator, making the preparation tedious, hampering the possibility to add it in a microfabrication process for a high-volume cantilever production.

In this work, we demonstrate that coating the cantilevers with amorphous carbon (a-C) overcomes the problems resulting from the above cited methods. a-C displays low reflectivity in water at $\lambda$ = 785 nm: a-C refraction index is $n_{2}$ = 2.32^31^, resulting in a *R* = 0.075. Thus, most of the laser power is transmitted to the a-C layer (92.5%), allowing it to be transformed into heat. Furthermore, it has an absorption coefficient around $\alpha$ ≈ 10^5^ cm^−1^, which means that after around 100 nm two thirds of the laser power is already absorbed by the a-C coating layer. Therefore, a few hundred nanometers will be enough for a complete harnessing of the laser excitation power into cantilever oscillation. a-C thin films exhibit also long-term stability in liquids, they are not toxic for biological materials, and their microfabrication process is robust, reproducible and integrable in a cantilever mass production. In the next sections, we present the potential of a-C thin films for photothermal excitation efficiency enhancement of AFM cantilevers.

## Results and discussion

Our aim was to enhance the photothermal cantilever excitation efficiency by means of a photothermal conversion layer (PCL) made of a-C. The a-C deposition on cantilevers was performed by the electron beam deposition (EBD) technique. Analyses performed with the energy-dispersive X-ray spectroscopy (EDXS) on an a-C layer deposited on silicon (111) show that its chemical composition is carbon, presenting a purity higher than 97% (more details in the Supplementary Information). We demonstrate the improvement in cantilever excitation efficiency with two types of commercially available gold coated cantilevers, with nominal spring constants of 26 and 85 N/m (MikroMasch Opus 160AC-NG and Olympus OMCL-AC55TS), proving the high stability of the PCL layer in liquid environment by imaging collagen and mica with atomic resolution in PBS solution.

**Figure 1:**
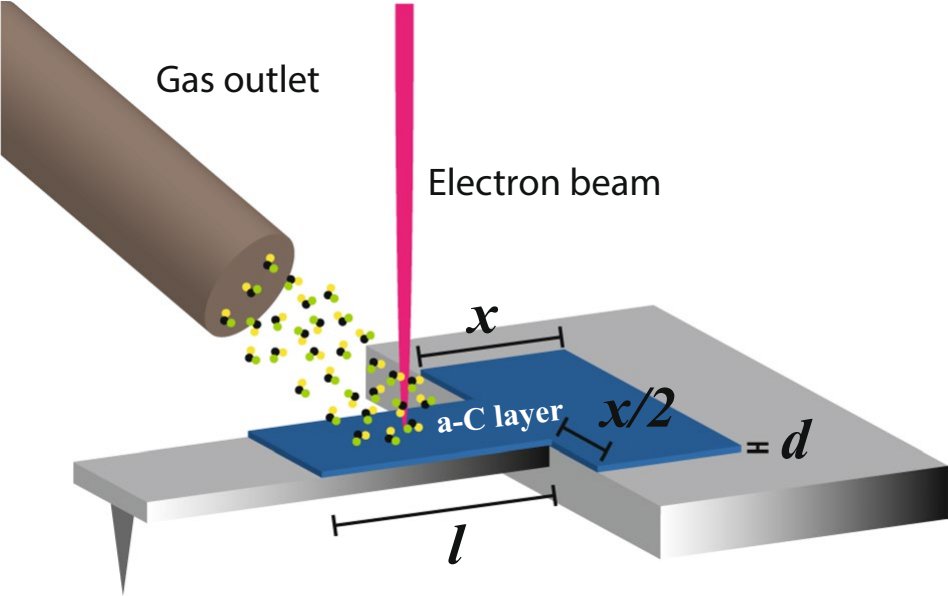
Schematic of the EBD coating process, where the gas precursor (naphthalene) is injected onto the substrate surface through a nearby gas-injection system, and dissociated by the electron beam irradiation, producing a deposited film on the same area of the scanned beam.

Figure 1 shows the a-C coating process on cantilevers by EBD. The gas precursor (naphthalene) is injected onto the substrate surface through a nearby gas-injection system, and dissociated by the electron beam irradiation, producing a deposited a-C film (blue on the image) on the same area scanned by the beam, controlling the thickness by setting a specific current dose. We performed the photothermal cantilever excitation enhancement study by optimizing the length (*l*) and thickness (*d*) of the PCL, and by tuning the PCL coating around the cantilever clamping area. The surface coated around the clamping area is shaped as a rectangle, whose short side measures *x*, while its long side measures *x* plus the cantilever width. We measured cantilever resonance spectra before and after the PCL coating to study the effect of the a-C film on the photothermal excitation efficiency, maintaining the same set up parameters (laser power and laser spot position). From now on, the cantilever excitation enhancement ratio is defined as follows:

$$ \eta = {\raise0.7ex\hbox{${A_{coated} }$} \!\mathord{\left/ {\vphantom {{A_{coated} } {A_{uncoated} }}}\right.\kern-\nulldelimiterspace} \!\lower0.7ex\hbox{${A_{uncoated} }$}} $$

where A_coated_ and A_uncoated_ is the maximum oscillation amplitude of the cantilever’s resonance curve after and before the PCL coating, respectively.

### Opus 160AC cantilever

**Figure 2:**
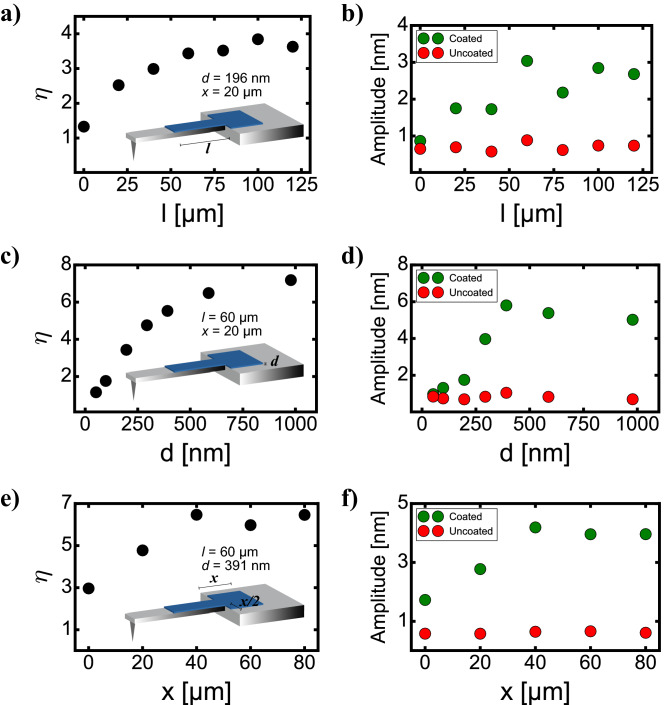
Enhancement ratio (left column) and cantilever’s oscillation amplitude (right column) before and after a-C coating on an Opus 160AC cantilever, depending on the coating length (**a**,**b**), film thickness (**c**,**d**) and clamping area deposition (**e**,**f**).

First, we performed a study on Opus 160AC-NG cantilevers. For time-saving purposes, we did not perform a parallel full optimization of *l*, *d* and *x*, but a sequential one, optimizing first the PCL coating length *l*, then the PCL thickness *d*, and finally the clamping area deposition *x*. As a first approximation, we consider that *l*, *d* and *x* are independent parameters, neglecting the effect on each other. We started the PCL length *l* optimization along the cantilever, increasing from 0 µm (no coating over the cantilever) to 120 µm. For the PCL length analysis, the thickness was kept constant at *d* = 196 nm, and the clamping area parameter was *x* = 20 µm. Results are shown in Fig. 2a. At low coating length values, the enhancement ratio $\eta$ increases as the PCL coating length increases, reaching a plateau around *l* = 60 µm with an enhancement ratio $\eta$ between 3 and 4, consistent with the 75 µm radius of the excitation laser spot in this experiment (see “Methods” section). As the excitation laser beam is focused close to the cantilever clamping, where the conversion of laser power into heat is maximized^32^, the larger the distance from the clamping area where the laser spot is focused, the lower is the portion of the laser absorbed by the PCL. This explains why PCL lengths larger than 60 µm produce similar efficiencies since at long distances from the clamping there is no laser absorption. Furthermore, a fraction of the laser beam power will always hit the PCL before the clamping. Thus, even when the cantilever is only coated on the clamping area (*l* = 0 µm), $\eta > 1$: the PCL hit by the laser on the clamping extent increases the heat absorption, which is transferred to the underneath gold/silicon cantilever structure, increasing the bimetallic effect and, as a result, the cantilever oscillation amplitude. For a more direct vision of the ratio enhancement, the oscillation amplitude before and after coating is displayed in Fig. 2b, where the ratio $\eta$ plateau is more evident when *l *≳ 60 µm.

Later, we observed the effect of the PCL thickness. For this purpose, we used a PCL length *l* = 60 µm, a value that is already at the plateau of the optimized enhancement ratio. Higher values would just increase the a-C deposition time, leading to marginal increments of $\eta$. The clamping area parameter was kept at *x* = 20 µm. The results are plotted in Fig. 2c,d. As expected, a PCL thickness increase rises $\eta$, since a thicker a-C PCL absorbs a higher portion of the laser power, increasing the temperature of the cantilever structure and, therefore, inducing a stronger bimetallic effect. However, thicknesses larger than *d *≳ 500 nm do not lead to an appreciable rise in $\eta$, as after a few hundred nanometers the laser beam is totally absorbed by the PCL so no further $\eta$ increase is produced, as expected from the absorption coefficient value around $\alpha$ ≈ 10^5^ cm^-1^ mentioned in the introduction. This was also confirmed in optical experiments performed on 160AC cantilevers coated with around 500 nm of a-C, where the transmission, reflection and absorption values of the excitation laser on a a-C PCL were 1.1%, 9.3% and 89.6% respectively, close the low reflection we theoretically calculated above (7.5%), confirming that most of the excitation power is absorbed by the PCL (more details in the Supplementary Information). It is worth mentioning that the fact that maximum $\eta$ values are attained at thickness around *d *≈ 500 nm confirms that the physics behind the excitation enhancement are mainly related to laser heat absorption, not to the bimetallic effect between the PCL and the cantilever. In the case of the latter, we would expect the maximum enhancement when the thickness of the PCL matches the thickness of the cantilever, which has a nominal value of *d*_*cantilever*_ = 4 µm. Nevertheless, the maximum is reached when *d* = 500 nm, excluding the bimetallic effect as the underlying mechanism of the excitation enhancement.

Finally, we proceeded to maximize the PCL clamping area. With the intention of reducing the PCL coating time while keeping a high $\eta$, a PCL length *l* = 60 µm along the cantilever and a thickness of *d* = 392 nm was used for the clamping area maximization, which displays a $\eta$ around 6 and requires lower PCL deposition times. Results are shown in Fig. 2e,f. The enhancement ratio $\eta$ rises while the PCL area enlarges, until it reaches a plateau at around *x* = 40 µm. The PCL surface around the clamping absorbs some heat power from the laser, but as soon as *x* gets too large, most of the new PCL area is not exposed to the laser beam and, therefore, it does not absorb heat power from the laser, producing no effect on the enhancement ratio $\eta$. Thus, compromising coating time and high $\eta$ values, an optimal value for the coating clamping area is *x* = 40 µm resulting in $\eta$ = 6.46, which needs a deposition time of 3 h and 14 min. This long-time coating is unpractical for a daily basis in most laboratories. In such case, sputtering methods would be necessary to deposit the required thicknesses in a few minutes^33,34^ through photoresist masks directly on the microfabrication process of the cantilevers. The EBD technique used here is intended as a proof of concept to demonstrate the capability to enhance the photothermal efficiency excitation on a-C coated cantilevers.

In order to keep the cantilever force sensitivity after the PCL coating, the cantilever’s spring constant $k_{c}$, resonance frequency $f_{0}$ and quality factor $Q$ must not largely differ from their original values previous to the film deposition, since the minimum detectable force depends on them^35^:

$$ F_{min} = \sqrt {\frac{{4k_{c} k_{B} TB}}{{\pi f_{0} Q}}} $$

where $k_{B}$ is the Boltzmann constant, $T$ is the temperature and $B$ is the measurement bandwidth. We have calculated those parameters for every tested cantilever, but we did not find large changes in their values. For example, for the thicker deposition on the thickness study (*l* = 60 µm, *d* = 978.32 nm, *x* = 20 µm), cantilever’s parameters changed from $k_{c}$ = 27.4 N/m, $f_{0}$ = 125,516 Hz and $Q$ = 5.27 to $k_{c}$ = 29.7 N/m, $f_{0}$ = 131,088 Hz and $Q$ = 5.34 after the PCL coating, leading to an increase on the $F_{min}$ around 1.2%, which is neglectable for AFM experiments. Consequently, the use of the PCL for photothermal enhancement did not significantly decrease cantilever’s force sensitivity. As the a-C PCL is not homogenously deposited on the cantilever but only in some specific areas, some non-linear effects in the equivalent cantilever stiffness are expected. For that reason, we have performed thermal tunes of the cantilevers before and after the a-C PCL coating, calculating later the cantilever stiffness. We did not see noticeable changes on the thermal tunes or cantilever stiffnesses, dismissing large effects on the cantilever’s spring constant due to the non-homogeneous and non-linear effects of the a-C PCL.

### OMCL-AC55TS cantilever

**Figure 3:**
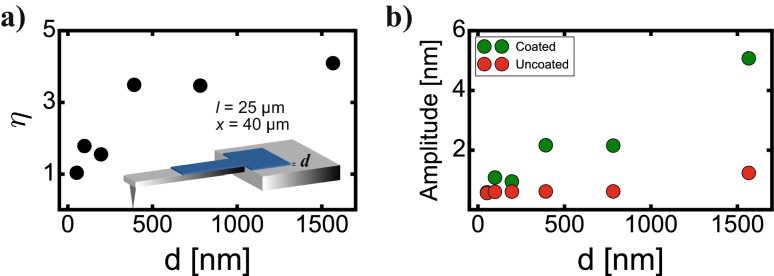
Enhancement ratio (**a**) and cantilever’s oscillation amplitude (**b**) before and after a-C coating on an OMCL-AC55TS cantilever, depending on the cantilever coating thickness.

In addition, we analyzed OMCL-AC55TS cantilevers, which are widely used in high-speed AFM in liquids due to their high resonance frequency. In this case, we only performed a thickness study. We coated a length *l* = 25 µm (cantilever length *l*_*cantilever*_ = 55 µm), leaving the area close to the tip uncoated, since it is there where the laser used for the oscillation detection is focused. The a-C film on the tip area would decrease the reflectivity (compared to the bare gold surface), hence decreasing the sensitivity of the beam deflection system. A rectangular surface of 120 × 40 µm^2^ was coated on the cantilever clamping area, which resulted from optimized value on Opus 160AC cantilevers presented in the last section. The results are displayed in Fig. 3a,b. Similarly to Opus 160AC cantilevers, increasing the thickness rises the enhancement ratio $\eta$, until a plateau is reached around 400 nm. This value is analogous to Opus 160AC cantilevers and much lower than the cantilever thickness (*d*_cantilever_ = 2.35 µm), reinforcing the idea that the main effect driving the photothermal excitation enhancement is the heat absorption instead of PCL/cantilever bimetallic effect, showing that after a few hundred nanometers the laser heat power is totally absorbed by the PCL. In this case, the maximum enhancement ratio is reached at *d* = 392 nm, resulting in $\eta$ = 3.49. As in the previous cantilever case, the *d* = 392 nm case requires long deposition times, 2 h and 50 min, which could be dramatically shortened using sputtering techniques through photoresist masks in the microfabrication process. We have also calculated $k_{c}$, $f_{0}$ and $Q$ for every tested cantilever, but as in the case of Opus 160AC cantilevers, we did not notice large changes in their values and therefore on the minimum detectable force.

The lower efficiency enhancement value of the OMCL-AC55TS cantilever ($\eta$ = 3.49) compared to the Opus 160AC cantilever ($\eta$ = 6.46) could be caused by multiple reasons. First, we did not optimize all the parameters in the case of the OMCL-AC55TS cantilevers, only thickness. In addition, both cantilever types have different lengths, thicknesses and cross-sections, which could affect the maximum reachable efficiency enhancement. Photothermal excitation efficiency and optimal irradiation positions of an excitation laser are affected by the three-dimensional shapes of cantilevers^25^. Future theoretical studies are needed in order to investigate the effect of the cantilever shape on $\eta$. Finally, experimental errors in the coating thickness, cantilever calibration, spot laser position, etc. could also slightly affect $\eta$ values.

### Stability of PTC layers and AFM imaging in liquids

**Figure 4:**
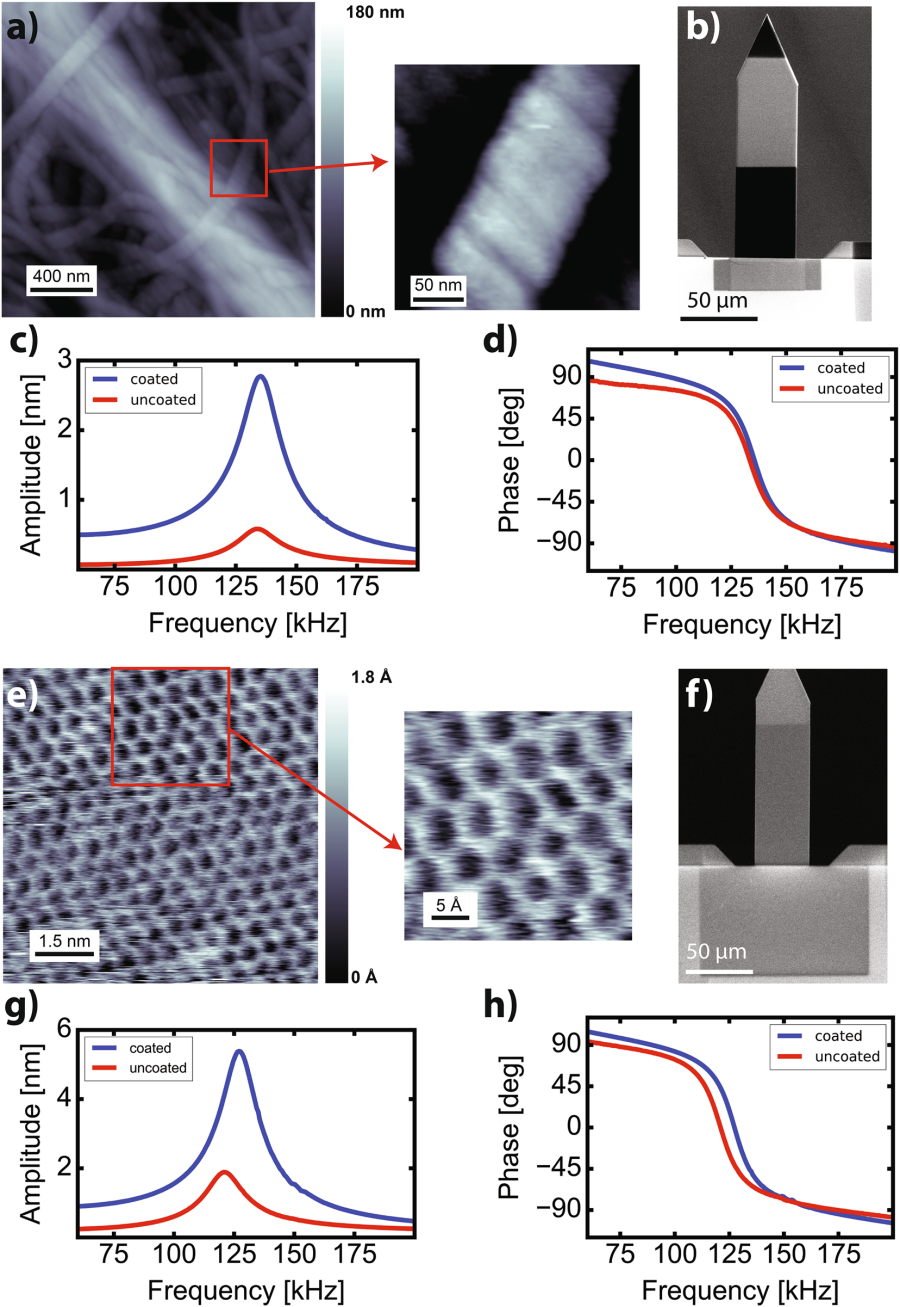
FM-AFM images of (**a**) collagen matrix and (**e**) mica surface in PBS solution. The inset in the images corresponds to a zoomed area enclosed by the red squares. In each case, an SEM image of the coated cantilever used on the measurements of collagen (**b**) and mica (**f**) is shown, as well as the oscillation amplitude versus frequency (**c**,**g**) and phase versus frequency (**d**,**h**) curves before (red) and after (blue) the a-C coating, exhibiting spectra free from spurious resonances.

The stability and water corrosion resistance of the PCL in liquid environments is crucial for a stable operation of the photothermal excitation system in dynamic-mode AFM. Also, it is important to maintain the tip radius after the PCL deposition to avoid a reduction of cantilever’s tip spatial resolution. To demonstrate the measurement capabilities of the cantilevers after a-C coating, we have measured collagen matrix from osteoblasts cells and mica in PBS solution. We have imaged the extracellular collagen matrix produced by osteoblasts with one of the Opus 160AC cantilevers used on the PCL clamping area study (*l* = 60 µm , *d* = 392 nm, *x* = 20 µm, $\eta$ ≈ 5) with the frequency modulation AFM method (FM-AFM), as shown in Fig. 4a. Measuring collagen presents some challenges. Since the surface roughness is several hundreds of nanometers, the tip may snap into the surface if the cantilever oscillation amplitude is not large enough and if the cantilever stiffness is low. In case of using photothermal excitation, high excitation efficiency is required to reach enough oscillation amplitudes with stiff cantilevers, hence avoiding high laser power that could damage and heat up the sample. As depicted in Fig. 4a, the image exhibits stable imaging condition and high resolution, able to distinguish the typical 67 nm periodicity of collagen D bands, as displayed on the zoom area inset enclosed on the red square. Also, a scanning electron microscope (SEM) image of the coated cantilever used on the measurements is shown in Fig. 4b, where the darker area of the a-C PCL area is clearly distinguishable. The dark area contrast at the cantilever’s end is due to the partial gold backside coating of the as purchased Opus 160AC-NG cantilevers, corresponding to the cantilever’s backside area that is not gold coated. Figure 4c displays oscillation amplitude versus frequency curves of the cantilever before (red) and after (blue) the a-C coating, presenting a clean resonance peak without influence from any spurious resonances, and how the amplitude is enhanced after the a-C coating; the phase versus frequency curve (Fig. 4d) is also free from spurious peaks, exhibiting the expected (− 90, + 90) degrees range.

In order to demonstrate that atomic resolution can be achieved after PCL deposition, we have imaged the atomic structure of mica in PBS solution with an a-C coated cantilever. To check if the coating process affects the cantilever’s tip resolution, we have deposited on an Opus 160AC cantilever a thick layer of a-C, and a large area on the cantilever and clamping areas (*l* = 100 µm, *d* = 1956 nm, *x* = 40 µm, $\eta$ ≈ 5.5). An example of an FM-AFM image of a mica surface is shown in Fig. 4e, where the atomic honeycomb-like patterns are easily distinguishable, more visible on the zoom area inset enclosed on the red square. An SEM image of the cantilever after PCL deposition is also displayed in Fig. 4f, as well as amplitude versus frequency (Fig. 4g) and phase versus frequency (Fig. 4h) curves before (red) and after (blue) the a-C coating, exhibiting spectra free from spurious resonances. Long-term stability was also investigated, where cantilever’s oscillation amplitude versus frequency curves were observed for 6 h (see Supplementary Figure S3). Changes on the photothermal excitation response were not detected, the oscillation amplitude remained constant, and the PCL did not display any sign of corrosion or degradation. These results reveal that the photothermal excitation efficiency is extremely stable in liquids.

## Conclusions

In this study, we have reported a method to enhance the photothermal excitation efficiency in dynamic-mode AFM, using an PCL made of a-C, coated by the EBD technique on an SEM. The a-C PCL increases the absorption of the laser power, reducing the laser reflection on the cantilever surface compared to a bare gold coated cantilever. We have performed a systematic study on an Opus 160AC cantilever to find the optimum parameters (PCL length *l* and thickness *d*, and clamping *x* coated area) to achieve a high enhancement ratio $\eta$ while keeping a low coating process time. We found the optimal values to be *l* = 60 µm, *d* = 392 nm and *x* = 40 µm, attaining an enhancement of $\eta$ ≈ 6.46. In the case of OMCL-AC55 cantilevers, using *l* = 25 µm, *d* = 392 nm and rectangular surface of 120 × 40 µm^2^ on the clamping area, a $\eta$ = 3.49 was attained. As $k_{c}$, $f_{0}$ and $Q$ values do not largely differ from their original values after PCL deposition, changes in the minimum detectable force can be neglected, and therefore the cantilever’s force sensitivity does not degrade. Although the required long-time coating is unpractical for a daily basis in most laboratories, the a-C deposition can be directly implemented in the cantilevers microfabrication process through photoresist masks using sputtering deposition techniques, which could deposit several hundreds of nanometers in a few minutes. However, in our proof of concept study presented here, the a-C PCL provides more than six-fold improvement in the excitation efficiency for a standard Opus 160AC cantilever, extremely useful for oscillating stiff cantilevers in liquid environments using low power excitation laser to prevent sample heating and damage. We demonstrated that cantilevers with PCL can be used to measure rough surfaces such as collagen matrix from osteoblasts or can achieve atomic resolution as depicted on the honeycomb-like patterns on mica surfaces. Furthermore, we demonstrated the long-term stability of the a-C coating, scanning mica surfaces for hours keeping the high photothermal excitation efficiency and the atomic spatial resolution. The high excitation efficiency and spatial resolution combined with the long-term coating stability will help to extend and broaden the use of the photothermal excitation for AFM in liquid environments, especially on fragile biological samples.

## Methods

a-C film deposition on cantilevers was performed by the EBD technique, in a Helios G4 CX Dual Beam system from FEI (Thermo Fisher Scientific, USA). For the cantilever coating, the focused electron beam current was set to 0.18 µA at 15 kV acceleration voltage, with a working distance of 4 mm. Naphthalene (C_10_H_8_) was used as gas precursor, injected onto the substrate surface through a nearby gas-injection system (GIS), and dissociated by the electron beam irradiation, producing a deposited film on the same area of the scanned beam. The thickness is controlled by means of setting a specific current dose, which was previously calibrated with the AFM, mimicking the deposition rectangular shape areas on a flat silicon surface.

Energy dispersive X-ray spectrometry (EDXS) experiments were carried out with an X-ray detector X-MaxN 50 (Oxford Instruments, UK) mounted on a Helios G4 CX Dual Beam system from FEI (Thermo Fisher Scientific, USA). EDXS spectra were collected with the electron beam at the acceleration voltage of 5 kV, and a beam current of 1.4 nA.

a-C PCL optical properties were studied by measuring the transmittance, reflectance and absorptance of the excitation laser on the a-C thin films deposited on silicon cantilevers (AC160-TN, Olympus) by the EBD technique. The infrared laser ($\lambda$ = 785 nm, power = 18 mW) was irradiated on the PCL, measuring the transmission and the reflection power by means of a laser power meter Q8230 (ADCMT). Finally, the absorptance of the infrared laser was calculated by subtracting the measured transmitted and reflected powers to the input laser power.

Primary osteoblastic cells were obtained from the calvarias of 1-day old C57BL/6 mice, as previously described^36^. Briefly, calvarias were resected and placed into ice-cold PBS to remove the surrounding tissue, and digestion of the calvarias with collagenase/dispase (sigma-Aldrich) in PBS solution for 10 min repeated four times. The cells obtained from each digestion except the first fraction plated in complete medium MEMα (Gibco) containing 10% FBS and 100 units/ml of penicillin and 0.1 g/ml streptomycin and grown at 37 °C with 5% CO_2_. Adherent osteoblasts harvested after three days with trypsin/EDTA. Osteoblasts were cultured in standing thin silicon nitride (SiN_x_) membranes with holes (Norcada NH050D549, thickness = 200 nm, hole diameter = 5 µm, pitch = 10 µm). First, osteoblasts were cultured on one side of the membrane and then, the structure was flipped upside-down to measure the collagen matrix produced on the basal side of the cells and spread through the membrane holes. The collagen matrix and osteoblast cells were fixed with formaldehyde for 1 h prior to the measurements, which were measured in PBS solution. The animal procedures were in accordance with the standards set forth in the Guidelines for the Care and Use of Laboratory Animals at the Takara-machi campus of Kanazawa University, Japan. This study was approved by the regional ethics committee (Medical Ethics Committee of Kanazawa University, No. 194073).

AFM experiments were carried out with a custom-built AFM, equipped with an ultra-low noise cantilever deflection sensor^37,38^. The cantilevers were driven by photothermal excitation with an infrared laser beam with a wavelength at $\lambda$ = 785 nm^39,40^. The excitation laser was focused on the cantilever clamping area, always maximizing the cantilever oscillation amplitude. The excitation laser beam was focused by means of 5 × and 10 × objectives on Opus 160AC and OMCL-AC55TS cantilevers, respectively, resulting in a diameter of the excitation laser spot of 150 µm for Opus 160AC cantilevers and 50 µm for OMCL-AC55TS cantilevers. For all the cantilever’s resonance spectra performed in Figs. 2 and 3, and for the ones displayed in Fig. 4c,g, the average laser power (*P*_*0*_) was set at 10 mW and the laser power modulation amplitude (*P*_*m*_) at 7.77 mW. Resonance spectra before and after a-C coating were measured in miliQ water to avoid any contaminant on the cantilevers' surface prior to a PLC deposition.

FM-AFM mode was used for imaging. The AFM scanning was controlled by a commercial SPM controller (ARC2, Asylum Research) and the oscillation amplitude (*A*) of the cantilever was kept constant using a commercially available controller (OC4, SPECS). The AFM was operated in constant frequency shift ($\Delta f$) mode, where the tip-sample distance is adjusted so that $\Delta f$ is kept constant. For AFM imaging, Opus 160AC-NG cantilevers from MikroMasch with a nominal spring constant of 26 N/m were used. Collagen images (256 × 256 pixels) and mica (512 × 512 pixels) were obtained in PBS solution. The AFM control parameters used for collagen were $\Delta f$ = 292.95 Hz, *A* = 1.8 nm, scan rate = 2 Hz and *P*_*m*_ = 4.66 mW; while for mica were $\Delta f$ = 1.17 kHz, *A* = 0.24 nm, scan rate = 9.77 Hz and *P*_*m*_ = 0.432 mW. In the case of mica imaging, the cantilever was previously coated with 15 nm of silicon to increase stability in liquid environments^41^. We note that the individual spring constant of each cantilever was also calibrated using its thermal noise spectrum^42^. Cantilever resonance curve processing and $\eta$ enhancement calculations were carried out with Python. Image processing was performed by using the WSxM image analysis software.

## Supplementary information

Supplementary Figures.
