# Supplementary material for: Photothermal excitation efficiency enhancement of cantilevers by electron beam deposition of amorphous carbon thin films
Source: Sci Rep. 2020 Oct 15;10:17436. doi: 10.1038/s41598-020-74433-x (PMC7562866; doi:10.1038/s41598-020-74433-x)
Supplement: Supplementary file 1 — Supplementary Figures. [file 41598_2020_74433_MOESM1_ESM.docx]

**Photothermal excitation efficiency enhancement of cantilevers by electron beam deposition of amorphous carbon thin films: Supplementary Information**

**Marcos Penedo**1,***, Ayhan Yurtsever**1**, Keisuke Miyazawa**1,2,3**, Hirotoshi Furusho**1**, Kiyo-Aki Ishii^4^, Takeshi Fukuma**1,2,3*

1Nano Life Science Institute (WPI-NanoLSI), Kanazawa University, Kanazawa 920-1192, Japan

2Division of Electric Engineering and Computer Science, Kanazawa University, Kakuma-machi, Kanazawa 920-1192, Japan

^3^Faculty of Frontier Engineering, Kanazawa University, Kakuma-machi, Kanazawa 920-1192, Japan

^4^Department of Integrative Medicine for Longevity, Graduate School of Medical Sciences, Kanazawa University, Kanazawa 920-8640, Japan

*fukuma@staff.kanazawa-u.ac.jp, marcos.penedo@staff.kanazawa-u.ac.jp


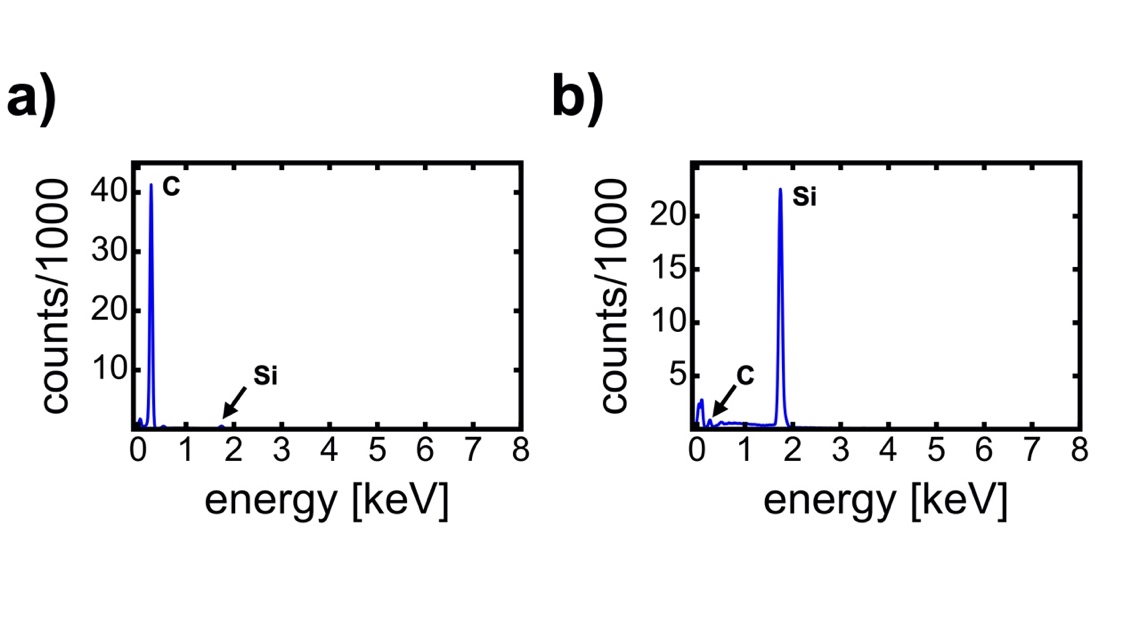
**Energy-dispersive X-ray spectroscopy experiments (EDXS) on a-C and silicon (111)**

**Figure S1 EDXS measurements on a-C and silicon (111)**. a) X-ray spectrum of a-C deposited on silicon (111) by the EBD technique. The deposited area was a 1 µm x 1 µm square, with an a-C coating thickness of around 1 µm. b) X-ray spectrum of an uncoated silicon (111) surface.

In the Figure S1a, a main peak at 0.27 keV can be distinguish, which corresponds to the carbon contained in the a-C coating. A secondary peak at 1.74 keV can be identified, much smaller than the carbon one, which corresponds to silicon, produced by the silicon substrate used to grow the a-C layer. The calculated material content ratio at the a-C coated surface are 97.02 % ± 0.10 % of C and 2.98 % ± 0.10 % of Si. It is worth mentioning that there are no other peaks on the graph, excluding the presence of contaminants. As a control, we have performed EDXS measurements in uncoated silicon, a few micrometers apart from the a-C deposited square. The results are displayed in the Figure S1b. The peak of silicon at 1.74 keV is much higher than the peak of carbon at 0.27 keV, whose origin might be contaminants at the surface with high amount of carbon content. In this case, the calculated material content ratio at the uncoated Si surface are 3.90 % ± 0.14 % of C and 96.10 % ± 0.14 % of Si. Thus, we confirm that we are depositing a high content carbon layer on the cantilevers by the EBD technique, and no other unwanted materials. The peaks below 0.27 keV are due to background noise. EDXS spectra were collected with the electron beam at the acceleration voltage of 5 kV, and a beam current of 1.4 nA.

**Optical properties of a-C PCL on AC160-TN cantilevers**


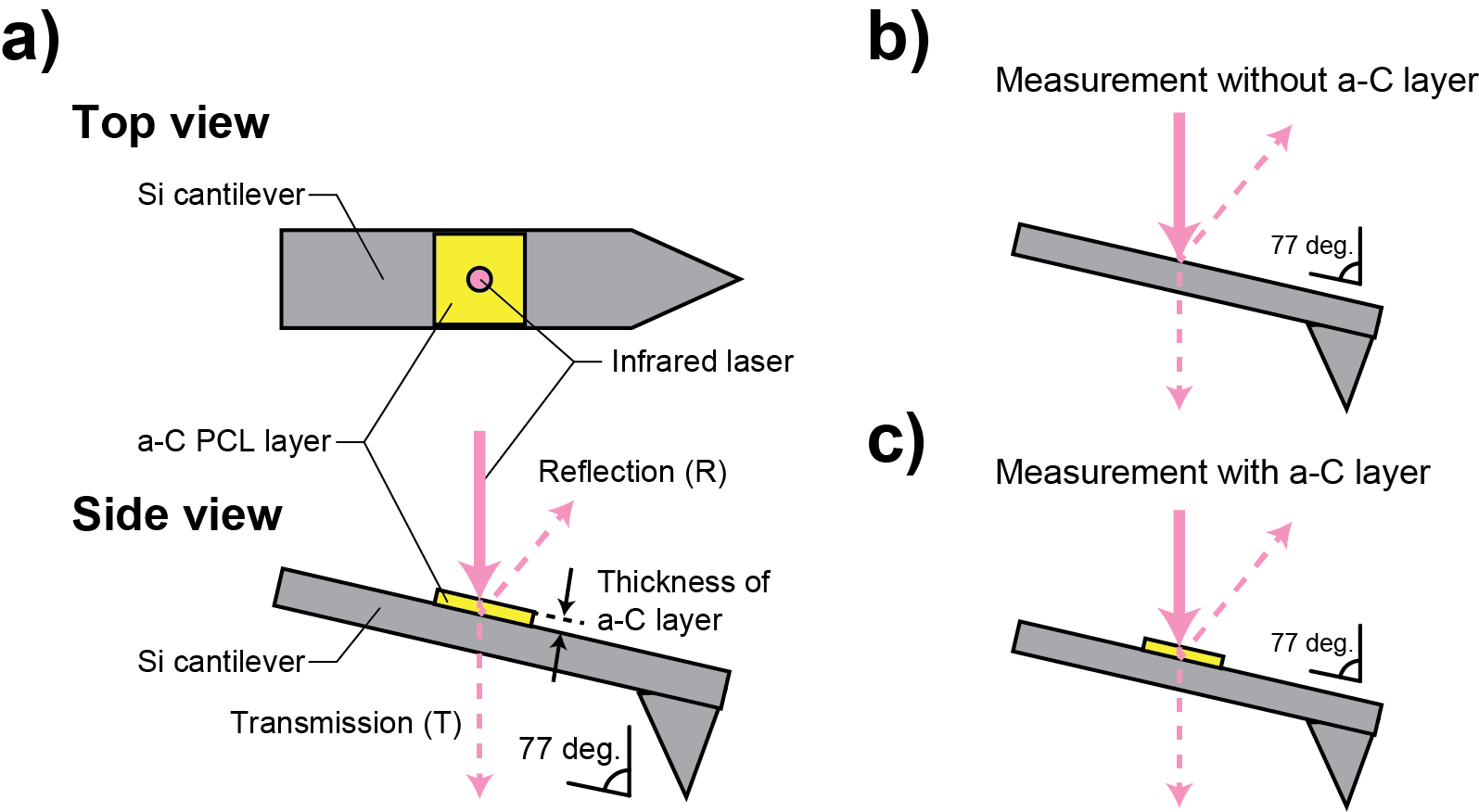


**Figure S2**. **Optical properties of a-C PCL**. a) Schematic of the experimental setup used to measure the optical properties of the a-C PCL, displaying top and side views. Measurements were performed at the center of the cantilever without b) and with c) the a-C PCL.

We have studied the laser ($\lambda$ = 785 nm, power = 18 mW) transmission (T), reflection (R), and absorption (A) in a-C films deposited on silicon cantilevers (AC160-TN, Olympus), as displayed in the schematic of the Figure S2a. We irradiated the infrared laser with an angle of 77 degrees respect to the cantilever plane, as in system we used for the AFM measurements. First, we focused the infrared laser at the center of the uncoated cantilever backside, as shown in Figure S2b. The optical measured values were:

- Uncoated silicon cantilever: T = 33.3 ％, R = 31.4 ％ and A = 35.3 %

Second, we coated the center of the cantilever backside (square area 40 x 40 µm^2^) with a-C by the EBD technique, focusing the infrared laser on that area, as depicted in Figure S2c. We have deposited two different thicknesses of the a-C PCL, 119 nm and 473.0 nm. The resulted optical values for the different cantilever coating thicknesses were:

- Cantilever coated with 119 nm of a-C: T = 12.3 %, R = 13.8 % and A = 73.9 %
- Cantilever coated with 473.0 nm of a-C: T = 0.3 %, R = 9.3 % and A = 90.4 %

Finally, we calculated the absorption of the infrared laser on the a-C film itself after subtracting the components of the silicon cantilever from the obtained results. The obtained final measurement results for both thicknesses were:

- 119 nm of a-C: T = 36.8 %, R = 9.5 % and A = 53.7 %
- 473.0 nm of a-C: T = 1.1 %, R = 9.3 % and A = 89.6 %

These results demonstrate that a-C PCL has a low reflectivity, and a-C thicknesses higher than 500 nm already absorb more than 90 % of the laser power, which dramatically increases the photothermal excitation efficiency.

**Stability of PTC layers in liquids**





**Figure S3**. **Long-term PCL stability in PBS solution**. Cantilever’s oscillation amplitude versus frequency curves acquired with the same cantilever shown in Fig.4f of the main manuscript. For 6 hours scanning, changes on the photothermal excitation response were not observed, the oscillation amplitude remained constant, and the PCL did not display any sign of corrosion or degradation. These results demonstrate that the photothermal excitation efficiency is extremely stable in PBS solutions. The laser power modulation amplitude (*P_m_*) was set at 7.77 mW during this experiment.
